# Reproductive performance of a semi-automated insemination system technology in sows

**DOI:** 10.1590/1984-3143-AR2025-0146

**Published:** 2026-06-05

**Authors:** Kebb Borstnez, Janine Camargo, Maiko Giorgi Philippe, Jean Carlo Volpato Faccin, Vanessa Peripolli, Ivan Bianchi, Ricardo Zanella, Mariana Groke Marques

**Affiliations:** 1 Programa de Pós-graduação em Produção e Sanidade Animal, Instituto Federal Catarinense – IFC, Araquari, SC, Brasil; 2 Programa de Pós-graduação em BioExperimentação, Escola de Ciências Agrárias Inovação e Negócios, Universidade de Passo Fundo – UPF, Passo Fundo, RS, Brasil; 3 Instituto Federal Catarinense – IFC, Araquari, SC, Brasil; 4 Embrapa Suínos e Aves, Concórdia, SC, Brasil

**Keywords:** swine reproduction, management, pig farming, breeding efficiency, labor optimization, litter size

## Abstract

Artificial insemination (AI) is a key biotechnology applied to disseminate superior genetics; however, its success depends on skilled labor to ensure accurate and consistent execution. A semi-automated insemination system (SAIS), capable of controlling semen deposition and reproducing the pressure of natural mating, can align AI procedures more closely with the sow’s physiological responses. The objective of this study was to evaluate whether the use of SAIS in post-cervical AI can improve reproductive efficiency in sows. A total of 449 females were inseminated, with 228 sows assigned to SAIS and 221 to the conventional method (CONV). Groups were balanced for parity, body condition, inseminator, and boar distribution. Sperm quality during storage did not differ between treatments. However, significant differences emerged during AI procedures: the SAIS group required less time to perform AI (18.60 ± 5.65 vs. 22.52 ± 12.38 seconds; P < 0.0001) and presented a lower incidence of semen backflow after AI (43.28% vs. 66.15%; P < 0.0001). The backflow negatively affected conception rate (P = 0.0342), although it did not influence total or live-born piglet numbers. Regarding reproductive performance, conception and farrowing rates were similar between groups. Nevertheless, females inseminated with SAIS have a higher total number of piglets born (16.97 ± 3.38 vs. 15.70 ± 3.91; P = 0.0027) and more live-born piglets (15.79 ± 3.3 vs. 14.46 ± 3.6; P = 0.0016) compared with CONV. Since backflow was not associated with litter size, this improvement cannot be attributed solely to reduced reflux. Instead, factors such as more standardized semen deposition and lower variability between inseminations may have contributed to this improvement. In conclusion, SAIS proved to be a practical and advantageous tool for AI in sows. Its use reduced time, decreased backflow, and increased litter size, representing a promising strategy to enhance efficiency in commercial herds.

## Introduction

Modern swine production has undergone significant advancements in recent decades, establishing itself as one of the most innovative sectors within the animal agriculture industry. Among the most notable improvements is the substantial enhancement in reproductive performance of sows, particularly over the last decade, with emphasis on the increase in the number of piglets weaned per sow per year (PSY). Recent data report an average of 30.05 PSY, with top-performing systems reaching up to 37.79 PSY (Bortolozzo et al., 2015, 2023; AGRINESS, 2023). These results have been made possible through genetic improvement, improved herd health stability, and the widespread adoption of artificial insemination (AI) as a central reproductive biotechnology (Knox, 2014).

In major pork-producing countries, the adoption rate of AI ranges between 90% and 100%, underscoring its strategic importance in intensive swine production systems (Lopez Rodriguez et al., 2017; FAO, 2021; Mellagi et al., 2023). AI plays a crucial role not only in the efficient utilization of genetically superior boars but also in increasing the number of doses per ejaculate, which directly contributes to genetic progress and productivity (Robinson and Buhr, 2005).

In recent years, technologies aimed at enhancing the quality of insemination doses have assumed a central role in optimizing reproductive outcomes (Pezo et al., 2019a). Additional innovations such as the identification of subfertile boars, the development of more effective extenders and catheters, and the standardization of sow handling protocols have further enhanced the efficiency of swine production systems (Koketsu et al., 2017; Bortolozzo et al., 2023; Mellagi et al., 2023).

Despite these advancements, key challenges remain, particularly in relation to the technical training of personnel and the standardization of procedures during AI (Lucca et al., 2020). Efficient execution of AI demands skilled labor, and variability in inseminator performance may compromise outcomes, especially in large-scale systems (Sterle and Safranski, 1997; Fabino et al., 2017).

Against this backdrop, the pursuit of technologies that combine technical efficiency with reduced reliance on specialized labor has intensified (Sharifuzzaman et al., 2024). The goal is to incorporate tools that simplify procedures, promote standardization, and support both environmental and economic sustainability within the production system.

Among recent innovations is the development of semi-automated devices designed to enhance the consistency and efficacy of artificial insemination (AI). These systems are designed to mimic the frequency and pressure of natural mating, thereby aligning the insemination process with the sow’s reproductive physiology and standardizing the time required for the procedure. In the current context of seeking technology aligned with sustainable production outcomes, the implementation of systems such as the SAIS aims not only to standardize processes but also to promote increasingly technified and efficient swine reproduction management (Mahfuz et al., 2022; Marić et al., 2025).

Within this context, the present study aimed to evaluate the effectiveness of a semi-automated insemination system in maintaining semen quality and improving the reproductive performance of sows under intensive production conditions.

## Methods

### Experimental design

The present study was approved by the Institutional Animal Care and Use Committee (CEUA) under protocol number 377/2021. The trial was conducted on a commercial swine farm located in the municipality of Marechal Cândido Rondon, in the state of Paraná, Brazil (Latitude: 24° 33’ 21″ S; Longitude: 54° 03’ 25” W; Altitude: approximately 420 m). The farm operated as a farrow-to-finish system, housing a herd of 2,300 DB90 females (Landrace × Large White crossbred), which were maintained in facilities comprising both group pens and individual gestation stalls. The reproductive facilities were equipped with negative-pressure ventilation and a multi-phase drop feeding system (GSI®).

Over a 12-week experimental period, a total of 449 multiparous sows were inseminated. All females had a body condition score between 3 and 4 and no history of dystocia or reproductive disorders in the previous cycle. The sows were randomly allocated into two treatment groups: the semi-automated insemination system group (n = 228), inseminated using the semi-automated technology, and the control group (n = 221), inseminated using the conventional blister-pack method.

Group allocation was balanced according to parity order (PO), including animals from the 2nd to the 6th parity. To avoid experimental bias, weekly distribution ensured that both groups received a comparable number of sows within each parity class.

### Semen collection and processing

The semen doses used in this experiment were produced at an on-site collection and processing facility located within the same experimental unit in Marechal Cândido Rondon, Paraná, Brazil. The facility was equipped with a negative-pressure ventilation system and a multi-phase drop feeding system. The boar herd consisted of 12 males, aged between 12 and 36 months, from the LQ1250 and LI7600 genetic lines.

Semen was collected using the gloved-hand technique. The ejaculates were collected in 700 mL graduated thermal cups, which were pre-lined with non-spermicidal plastic to prevent sperm damage caused by sunlight exposure or thermal fluctuations.

Following collection, the ejaculates were subjected to both macroscopic (color, odor, and volume) and microscopic evaluations (total motility, concentration, and sperm morphology). Microscopic assessments were performed using the automated iSperm system. Sperm morphology was assessed using phase-contrast microscopy at 1000× magnification, with the evaluation of 100 spermatozoa per sample. The percentages of morphologically normal sperm, as well as those presenting abnormalities in the head, midpiece, tail, acrosome, proximal droplet, distal droplet, and total defects, were recorded according to the criteria described by Shipley (1999). Only ejaculates with total motility greater than 70% and less than 20% of the defects were used in the experiment.

Semen packaging was standardized to ensure homogeneity between experimental groups. For the semi-automated insemination system, semen was stored in 1-liter bags connected, at the IA moment, to a specialized gun (Pulse-flow® - GenePro), which allows controlled deposition of semen during the procedure. In contrast, the conventional group used 100 mL blister packs. In both treatments, the insemination dose consisted of 1.5 × 10^9^ sperm cells in a final volume of 45 mL, using a long-term extender. Each ejaculate was equally divided between the two treatments to avoid potential sire effects on the reproductive outcomes of the sows.

After packaging, insemination doses were stored for up to 48 hours at 17 °C in a semen-specific storage cooler. During transportation to the insemination site, all doses were maintained at a controlled temperature (17 °C) to preserve sperm integrity until use.

### Sperm quality during storage

Because semen was packaged in different types of containers according to the experimental groups, quality control was performed to ensure that storage conditions did not introduce bias in the comparative evaluation. For this purpose, total sperm motility and sperm morphology were assessed at pre-insemination, that is, after 48 hours of storage.

### Artificial insemination

To assess reproductive performance, females from the same contemporary group were selected following weaning. The sows were housed in individual gestation stalls and monitored twice daily (morning and afternoon) for estrus detection. The pre-insemination body condition score (BCS) was assessed using a Caliper device, ranging from 1 (the lowest BCS) to 5 (the highest BCS).

Estrus detection was performed with the aid of a sexually mature boar, which was walked in front of the gestation stalls to allow nose-to-nose contact with the females. Simultaneously, a technician applied manual back pressure to evaluate the standing reflex in the presence of the boar, known as the boar-induced standing reflex (BSR). Upon the first manifestation of BSR, females were inseminated following the farm’s fixed-time AI protocol: 0 h, + 24 h, and + 24 h.

Post-cervical artificial insemination (PCAI) was used in both treatments. Briefly, the insemination pipette was inserted at a dorsocranial angle into the vulvar vestibule. After fixation in the cervix, the inner catheter was carefully advanced into the uterine lumen to allow semen deposition in the anterior uterine body.

For the control group (CONV), a conventional blister pack was attached to the external end of the pipette and remained connected until complete semen release. In the semi-automated insemination system (SAIS), a semi-automated insemination device programmed to dispense 45 mL per dose was used. The device was connected to the pipette and activated via a button, initiating a controlled flow of the semen dose until entirely dispensed.

In both treatments, once the dose was emptied, the device or blister was disconnected, the pipette cap was closed, and the pipette remained inserted in the female until insemination was completed in a batch of five females. After each batch, the pipette was removed and discarded.

Importantly, all procedures throughout the experiment were carried out by the same trained technicians, who alternated between treatment groups to avoid operator-related bias.

### Evaluation of AI protocol duration and semen reflux

The insemination time per female was recorded in seconds using a digital stopwatch. Timing began immediately after the catheter was inserted into the vulva and ended upon complete absorption of the insemination dose.

Semen reflux was evaluated visually for up to 20 minutes post-insemination. Reflux was considered present when visible dripping of seminal content from the vulva was observed and recorded as a binary variable (presence/absence) for subsequent analysis. Also, semen backflow was analyzed for incidence, considering the total number of AI procedures (n = 1,376), and for reproductive performance, considering the total number of inseminated sows (n = 449).

### Reproductive performance

Return to estrus was monitored daily starting on day 18 after artificial insemination, using a sexually mature boar. Estrus detection followed the same procedure described previously, based on the boar-induced standing reflex.

Conception rate was determined via transabdominal ultrasonography (HIPER08®, China) conducted between days 25 and 35 after the last insemination. The rate was calculated as the number of pregnant females divided by the total number of inseminated females, expressed as a percentage, and then multiplied by 100.

Pregnant females were transferred to farrowing stalls at 108 days of gestation and remained there until parturition, which occurred at an average of 114 ± 7 days. Post-farrowing, the following variables were recorded: total number of piglets born, number of liveborn piglets, number of stillbirths, and number of mummified fetuses.

### Statistical analysis

Statistical analyses were performed using SAS software (SAS Institute, Cary, NC, version 9.4), with a significance level of 0.05%. Data normality was assessed using the Shapiro-Wilk test. For binary response variables (yes/no), the Chi-square test or Fisher's exact test was applied. Ordinal variables were analyzed using the Mann-Whitney test. Since none of the continuous variables followed a normal distribution, the Kruskal-Wallis test was used for mean comparisons, and the Chi-square test was used to evaluate differences in variances.

Semen backflow was evaluated using two distinct units of analysis: the incidence was calculated based on the total number of artificial insemination (AI) procedures performed (n = 1,376) to identify differences between experimental groups. Conversely, the impact of backflow on reproductive performance was analyzed per inseminated female (n = 449). Sows were categorized into the backflow yes group if they exhibited visible semen dripping in at least one of the three AI procedures, and into the backflow no group if no dripping was recorded across all procedures.

## Results

### Productive data before and after housing

The independent variables evaluated before and after housing were analyzed to verify the homogeneity of sow distribution between treatments (Table 1). No statistically significant differences were observed between groups for any of the parameters analyzed, indicating a balanced allocation of females across both treatments (P > 0.05).

**Table 1:** Reproductive and management-related variables of sows inseminated using the conventional artificial insemination (CONV) method and the semi-automated insemination system (SAIS), before and after housing.

**Variables**		**CONV**	**SAIS**	**P-value**
Parity order (mean ± SD; n females)		3.45 ± 1.23 (221)	3.31 ± 1.11 (228)	0.2931
Total piglets weaned from the previous litter		12.52 ± 2.57 (168)	12.00 ± 2.54 (173)	0.2040
(mean ± SD; n litters)				
Body condition score		3.12 ± 0.47 (221)	3.10± 0.42 (228)	0.4400
(mean ± SD; n females)				
Number of AI procedures		2.90 ± 0.31 (221)	2.89 ± 0.30 (228)	0.9274
(mean ± SD; n females)				
Inseminator % (n of inseminations)	1	44.35 (267/602)	48.18 (304/631)	0.2205
	2	47.67 (287/602)	42.00 (265/631)	
	3	0.17 (1/602)	0.16 (1/631)	
	4	7.81 (47/602)	9.67 (61/631)	
Boar used	1	6.87 (42/611)	8.04 (52/647)	0.8324
	2	0.16 (1/611)	0.15 (1/647)	
	3	2.62 (16/611)	2.16 (14/647)	
	4	1.96 (12/611)	3.09 (20/647)	
	5	0.16 (1/611)	0 (0/647)	
	6	0.49 (3/611)	0.77 (5/647)	
(n of doses)	7	19.15 (117/611)	19.94 (129/647)	
	8	16.20 (99/611)	13.45 (87/647)	
	9	3.76 (23/611)	2.78 (18/647)	
	10	23.57 (144/611)	25.19 (163/647)	
	11	7.69 (47/611)	8.19 (53/647)	
	12	12.60 (77/611)	12.21 (79/647)	
	13	4.75 (29/611)	4.02 (26/647)	

Significant difference for P < 0.05.

The average parity order was 3.45 ± 1.23 for the CONV group and 3.31 ± 1.11 for the SAIS group (P = 0.2931). The mean number of piglets weaned in the previous litter before housing was 12.52 ± 2.57 for CONV and 12.00 ± 2.54 for SAIS (P = 0.2040). The average body condition score (BCS) of females at the time of insemination was 3.12 ± 0.47 for CONV and 3.31 ± 1.11 for SAIS (P = 0.2931), values consistent with a good nutritional status in sows.

Throughout the experiment, a total of 1,376 artificial inseminations (AI) were performed: 706 in the CONV group and 670 in the SAIS group. The average number of AIs per female was 2.90 ± 0.31 for the CONV group and 2.89 ± 0.30 for the SAIS group, with no significant difference between the groups (P = 0.9274).

The distribution of AIs among inseminators was also homogeneous. Inseminator 1 performed 44.35% of the AIs in the CONV group and 48.18% in the SAIS group; Inseminator 2, 47.67% (CONV) and 42.00% (SAIS); and Inseminator 3 participated less in both groups. However, the analysis indicated no significant difference in inseminator distribution between treatments (P = 0.2205).

Regarding the origin of semen doses, boars 7, 10, and 12 were used most frequently, contributing 19.15%, 23.57%, and 12.60% of doses in the CONV group, and 19.94%, 25.19%, and 14.12% in the SAIS group, respectively. Despite differences among boars, the distribution of doses from the same boar between treatments was homogeneous (P = 0.8324).

### Sperm quality during storage

For the sperm quality parameters, no significant differences were observed between treatments. Total motility averaged 84.86 ± 7.66% in the CONV group and 86.14 ± 6.31% in the SAIS group (P = 0.5210). Similarly, the percentage of morphologically abnormal spermatozoa did not differ between groups, with values of 30.14 ± 13.35% in the CONV group and 30.91 ± 12.67% in the SAIS group (P = 0.4294) (Table 2).

**Table 2:** Sperm motility in the conventional artificial insemination (CONV) and semi-automated insemination system (SAIS) groups was evaluated after 48 hours of storage.

**Variables (% ± SD)**	**CONV**	**SAIS**	**P-value**
Total motility	84.86 ± 7.66 (118)	86.14 ± 6.31 (102)	0.5210
Abnormal morphology	30.14 ± 13.35 (170)	30.91 ± 12.67 (186)	0.4294

Significant difference for P < 0.05.

### Evaluation of AI protocol duration and semen reflux

The AI application time, measured in seconds, was shorter in the SAIS group (18.60 ± 5.65 seconds) compared to the CONV group (22.52 ± 12.38 seconds) (P < 0.0001). This indicates that the use of SAIS allowed for a shorter insemination time and greater standardization of the technique.

Regarding the incidence of semen backflow evaluated per procedure, the SAIS group presented a significantly lower percentage (43.28%; 290/670) within 20 minutes after AI compared to the CONV group (66.15%; 467/706) (P < 0.0001). When evaluating the impact of backflow per female, a lower conception rate was observed in sows that exhibited backflow in at least one AI procedure (86.40%; 235/272) compared to those without any backflow (93.22%; 165/177) (P < 0.05) (Table 3). However, this effect was not observed for the total number of piglets born or total live-born piglets.

**Table 3:** Influence of backflow on reproductive outcomes.

**Variables**	**CONV (n=706 AIs)**	**SAIS (n=670 AIs)**	**P-value**
Backflow Presence (%)	66.15%^A^ (467/706)	43.28%^B^ (290/670)	< 0.0001
Backflow Absence (%)	33.85% ^B^ (239/706)	56.72%^A^ (380/670)	
**Backflow**			
**Reproductive Outcomes**	**Yes (n = 272)**	**No (n = 177)**	**P-value**
Conception rate (%)	86.40% ^B^ (235/272)	93.22%^A^ (165/177)	0.0342
Live-born piglets (n)	15.00 (n=211)	15.31 (n=161)	0.8095
Total piglets born (n)	16.27 (n=211)	16.59 (n=161)	0.5330

A,B superscript letters indicate statistical difference between treatments. Significant difference for P < 0.05.

### Reproductive performance

Reproductive performance outcomes of sows inseminated under conventional management (CONV) and the semi-automated insemination system (SAIS) are presented in Table 4. Conception rate (87.33% in CONV vs. 90.79% in SAIS; P = 0.3076) and farrowing rate (86.43% vs. 89.04%; P = 0.3229) did not differ significantly between groups. However, sows inseminated with SAIS had higher litter sizes. The mean number of total piglets born was significantly greater in the SAIS group (16.97 ± 3.38) compared with CONV (15.70 ± 3.91; P = 0.0027). Likewise, the number of live-born piglets was higher in SAIS (15.79 ± 3.28) than in CONV (14.46 ± 3.84; P = 0.0016). No significant differences were observed for stillborn piglets (0.79 ± 1.08 in SAIS vs. 0.94 ± 1.39 in CONV; P = 0.7178) or mummified piglets (0.39 ± 0.65 in SAIS vs. 0.30 ± 0.58 in CONV; P = 0.1168).

**Table 4:** Reproductive performance of sows inseminated under conventional (CONV) management and with the semi-automated insemination system (SAIS).

**Variables**	**CONV (n)**	**SAIS (n)**	**P-value**
Conception rate (%)	87.33% (193/221)	90.79% (207/228)	0.3076
Farrowing rate (%)	86.43% (191/221)	89.04% (203/228)	0.3229
Total piglets born	15.70 ± 3.91A (179)	16.97 ± 3.38B (196)	0.0027
(mean ± SD)			
Total live-born piglets	14.46 ± 3.84A (180)	15.79 ± 3.28B (196)	0.0016
(mean ± SD)			
Stillborn piglets	0.94 ± 1.39 (180)	0.79 ± 1.08 (196)	0.7178
(mean ± SD)			
Mummified piglets	0.30 ± 0.58 (180)	0.39 ± 0.65 (196)	0.1168
(mean ± SD)			

(n) refers to the females evaluated. ^A,B^ superscript letters indicate statistical difference between treatments. Significant difference for P < 0.05.

## Discussion

Artificial insemination (AI) is the main reproductive biotechnology used in commercial swine production systems (Lopez Rodriguez et al., 2017; Maside et al., 2023). Therefore, the quality of the insemination dose and the reproductive management of the female, from estrus to the moment of AI, are essential factors for achieving satisfactory production indices in the swine industry (Mellagi et al., 2023; Lopez Rodriguez et al., 2017).

The production of insemination doses for swine is a highly specialized stage that has increasingly invested in technologies to automate the process and enhance efficiency in producing semen with standardized quality and adequate longevity until use in AI (Mellagi et al., 2023; Pezo et al., 2019b; Lopez Rodriguez et al., 2017). In this context, *in vitro* assessment of semen quality is a crucial tool for inferring the quality of the dose during production, storage, and subsequent use (Jung et al., 2015). Several studies investigating the incorporation of new products, such as extenders or changes in storage processes, use this *in vitro* assessment to validate the efficiency of products and processes (Camargo et al., 2025; Li et al., 2023; Menezes et al., 2016). Total and progressive sperm motility are widely used parameters in routine boar studs and experiments to evaluate semen quality (Jung et al., 2015).

Storage of diluted semen using SAIS technology proved to be an efficient alternative for maintaining boar sperm quality until AI, as it preserved both total motility and sperm morphology during the evaluated period. Swine semen is mainly marketed in refrigerated liquid form to ensure the preservation of semen dose quality (Viana et al., 2020). Homogenization or rotation of insemination doses during storage is recommended to resuspend sedimented sperm, improving cell contact with the substrates present in the extender (Belstra, 2007).

In the present study, the use of a single container with a volume higher than that of post – cervical doses (40-50 mL) did not impair sperm motility. However, pre-AI homogenization may have contributed to increased sedimentation. Although few studies have addressed the impact of this practice, Rodríguez-Gil and Rigau (1995) demonstrated that homogenization improves parameters such as sperm motility, acrosome integrity, and osmotic resistance.

Post-cervical artificial insemination (PCAI) does not require additional time for its execution, does not compromise animal welfare, and allows for a reduction in the number of sperm per dose, resulting in a significant increase in productivity (Knox, 2016). However, PCAI requires minimal staff training (Watson and Behan, 2002). Although it is a more complex technique due to the need for catheter passage to the initial intrauterine portion, PCAI has been widely used in sows (Viana et al., 2020). Training and experience of AI technicians significantly influence success rates and backflow minimization (Islam and Kumar, 2024; Knox, 2016).

Another relevant aspect of reproductive management is post-insemination backflow. Uterine backflow is frequently observed after mating or AI and is considered a physiological mechanism associated with the high endometrial contractility of females in estrus. This activity promotes sperm transport to the utero-tubal junction within approximately 30 minutes after insemination (Rath, 2002). However, despite being physiological (Langendijk et al., 2005), excessive backflow may compromise reproductive performance because it reduces the number of viable sperm available at the time of fertilization.

Backflow occurrence is influenced by several factors, such as the insemination site, the volume and viscosity of the inseminated dose, and the speed and pressure of semen deposition (Will et al., 2021; Waberski et al., 2019; Rath, 2002). Larger insemination volumes or rapid infusion into the uterus tend to increase the amount of semen lost, whereas slower deposition and reduced volume may minimize reflux (Will et al., 2021). It was also observed that the presence of backflow negatively influenced conception rate, which was higher in females without backflow, although it did not affect the number of total or live-born piglets.

Farm managers strive to standardize reproductive management to maximize production indexes and ensure sustainable production (Waberski et al., 2019). The use of SAIS technology enables shorter AI procedures and greater standardization of the technique, thanks to pressure and flow control for insemination volume deposition in the uterus. Also, automation technologies offer substantial potential for reducing labor requirements in swine production, addressing both rising labor costs and declining availability of skilled workers (Sun et al., 2024). In the present study, the semi-automated insemination system (SAIS) significantly reduced the time of AI and the incidence of post-AI backflow compared with conventional management. This effect can be attributed to the controlled pressure and pulse-flow technology, which mimics the frequency and flow pattern of natural mating. Such modulation of uterine stimulation may reduce the abrupt uterine contractions often triggered during conventional insemination, thereby decreasing the loss of semen.

Interestingly, females inseminated with SAIS also delivered larger litters compared with CONV. Since backflow was not associated with litter size, this outcome cannot be explained solely by the reduced reflux observed in the SAIS group. Other factors may have played a role, including a more consistent deposition of semen, improved alignment with the sow’s physiological responses, and reduced variability between inseminators. These aspects could lead to a more efficient use of sperm cells and better fertilization dynamics, which may explain the improved prolificacy observed in the SAIS group.

The implementation of semi-automated insemination systems (SAIS) aligns with the digital transformation of the pig industry (Mahfuz et al., 2022) and principles of Precision Livestock Farming (PLF) (Marić et al., 2025), where smart technologies are being applied to standardize management practices and enhance overall biological efficiency.

Finally, swine production has been seeking strategies to make the production system sustainable, and new technologies that facilitate management, promote standardization, and ensure profitability are essential for this goal. Furthermore, investing in staff training and continuous education enhances the quality control of techniques applied in farm routines (Marić et al., 2025; Mahfuz et al., 2022; Knox, 2016).

## Conclusion

The results of this study demonstrate that the semi-automated insemination system (SAIS) improves the efficiency of the insemination procedure and reduces semen backflow. Reduced backflow was associated with higher conception rates; however, the larger litter size observed in the SAIS group cannot be fully explained by this factor. It is likely that additional elements, such as greater consistency in semen deposition and reduced operator variability, contributed to the improved reproductive outcomes. Overall, SAIS represents a feasible and advantageous strategy for swine production, particularly for those facing challenges in hiring skilled labor, by providing a practical approach to artificial insemination.

## Data Availability

Data is provided within the manuscript. Other datasets analyzed in this study are available from the corresponding author upon reasonable request.
